# Taxifolin Modulates Transcriptomic Response to Heat Stress in Rainbow Trout, *Oncorhynchus mykiss*

**DOI:** 10.3390/ani12101321

**Published:** 2022-05-22

**Authors:** Irina V. Sukhovskaya, Nadezhda P. Kantserova, Liudmila A. Lysenko, Alexey A. Morozov

**Affiliations:** 1 Institute of Biology of the Karelian Research Centre of the Russian Academy of Sciences (IB KarRC RAS), 11 Pushkinskaya Street, 185910 Petrozavodsk, Russia; sukhovskaya@inbox.ru (I.V.S.); l-lysenko@yandex.ru (L.A.L.); 2 Limnological Institute of the Siberian Branch of the Russian Academy of Sciences (LIN SB RAS), 3 Ulan-Batorskaya Street, 664033 Irkutsk, Russia; morozov@lin.irk.ru

**Keywords:** taxifolin, heat stress, rainbow trout, liver, transcriptome, *Gyrodactylus* sp.

## Abstract

**Simple Summary:**

Cultivated rainbow trout face with multiple stressors that impact their viability, growth, and welfare. Heat stress provoked by summer temperature rise leads to increased mortality and accelerated transmission of bacterial and parasite pathogens in fish population. Feed supplements of plant-origin could alleviate stress in fish presumably via stimulation of their defense systems. To understand the mechanism of beneficial effects of taxifolin, an antioxidant extracted from larix wood, we compared transcriptomic responses to both temperature rise and flatworm infection in rainbow trout individuals fed either a standard ration or supplemented with taxifolin. Dietary taxifolin has been shown to mitigate some heat-induced responses in fish, such as biosynthesis of sterols, heat-shock proteins, and cell death regulators, while it does not interfere with temperature-dependent antioxidant induction. Interestingly, no transcriptomic response was induced due to *Gyrodactylus* infestation in trout, whereas parasite load was diminished due to heat treatment. Thus, the plant-origin supplementation of fish diet could be an easy way to improve the health of fish and promote their ability to tolerate stresses under intensive production.

**Abstract:**

Taxifolin is a natural flavonoid known for its antioxidant, anti-inflammatory, and antiproliferative effects on animals. In this work, we have studied the effect of this compound on rainbow trout, *Oncorhynchus mykiss*, a major object of aquaculture, under slowly increasing ambient temperature and *Gyrodactylus* flatworm infection. Transcriptomic profiling of liver samples performed by using the Illumina HiSeq 2500 sequencing platform shows that a combined taxifolin/heat treatment, unlike heat treatment alone, downregulates the production of isopentenyl diphosphate, likely affecting the production of cholesterol and other sterols. Taxifolin treatment also modulates multiple apoptosis regulators and affects the expression of HSPs in response to increasing temperature. On the other hand, the expression of antioxidant enzymes in response to heat is not significantly affected by taxifolin. As for the *Gyrodactylus* infection, the parasite load is not affected by taxifolin treatment, although it was lower in the high-temperature group. Parasite load also did not induce a statistically significant transcriptomic response within the no heat/no taxifolin group.

## 1. Introduction

Natural compounds such as alkaloids, terpenoids, flavonoids and polyphenols could be explored as a safe alternative of chemotherapeutics to control diseases in aquaculture species via increasing their resistance to environmental stressors and pathogens [1,2,3,4]. Those protective compounds may possess immunostimulating and antioxidant activities through delaying or preventing oxidative damage and disease vulnerability [5,6]. Although taxifolin, a bioflavonoid of plant origin, is widely used for food supplementation in human and animal nutrition [7], its beneficial effects in aquaculture species are scarcely reported [8,9]. Taxifolin has shown promise in treating, or at least reducing the markers of, a range of diseases and conditions [10,11]. Most obviously, it is an antioxidant and thus directly reduces the deleterious effect of ROS by radical scavenging. It was shown in vitro to reduce the activity of several radicals [12,13], as well as DNA damage inflicted by Fenton’s reagent [14]. Enzymatic antioxidant activity of cellular homogenates also increases in response to low doses of taxifolin, although with higher dosages, it seems to decrease again [8,14]. Besides direct radical scavenging, taxifolin also decreases the effect of ROS by inhibiting the activity of pro-oxidative enzymes such as myeloperoxidase, NADPH oxidase, cyclooxygenase, and nitric oxide synthase. For the latter two enzymes, decreased expression of corresponding genes along with decreasing activity of the enzyme itself were shown in mouse models with cerebral ischemia-reperfusion injury [15]. The same was shown for cytochrome P450 in chicken cells [16]. In human cell cultures, taxifolin was also shown to increase the expression of multiple cytoprotective and antioxidative genes, notably phase II detoxification enzymes; this effect was hypothesized to be mediated by Nrf2/ARE signaling [17], which was later confirmed by luciferase assay [18] and transient Nrf2 knockdowns [19].

The anti-inflammatory effects of taxifolin also involve decreasing the production of pro-inflammatory cytokines such as interleukin 1β (IL-1β) and tumor necrosis factor alpha (TNF-α) [20,21], likely by removing ROS or other radicals that serve as intermediary messengers in the inflammatory pathways [22]. There are also reports on taxifolin affecting lipid synthesis, cholesterol production, and ApoA/ApoB balance, though these effects are not caused by changes in transcription levels [23,24].

In addition to antioxidant and anti-inflammatory effects, taxifolin inhibits the proliferation of multiple cancer cell lines via activating pro-apoptotic mechanisms [25] and affecting cell cycle regulators including the Wnt/β-catenin signaling pathway [26] causing cell cycle arrest [27]. At the same time, the decrease in viability of (non-cancer) Vero cells is not statistically significant [25], and the absence of cytotoxic effects on human RPE cells within the range of studied concentrations (up to 100 μg mL^−1^) was also noted by Xie et al. [19].

In contrast, a number of studies on fish and fish cell lines detect taxifolin cytotoxicity for non-cancerous cells. A work on jointly exposing *Danio rerio* embryos to taxifolin and cadmium shows that, while lower concentrations of taxifolin protect from cadmium toxicity, excessive taxifolin (100 μM) even enhances the toxic effects of cadmium in terms of morphological deformity rate [14]. Taxifolin concentrations above 1 μM decrease the viability of carp hepatocytes in culture [28]. Awad et al. [8] also show that increasing concentrations of taxifolin lead to reduced effects in multiple immunological parameters, with 0.1% or less dietary taxifolin being the most beneficial concentration for gilthead seabream.

The importance of environmental temperature in determining the physiology and growth of fish is well-known [29,30,31]. Juvenile rainbow trout thermal optimum is 10–14 °C, and ambient temperatures exceeding their upper thermal limit (26 °C) inhibit whole-body growth, protein synthesis, and immunity in trout [32]. Increasing lethality over 28 °C, naturally occurring in late summer, results in substantial losses for trout aquaculture stock. Due to the high economic value of the species, their physiological mechanisms to tolerate heat stress should be studied in detail based on transcriptomic approach. Studies of trout transcriptome response to heat stress have been performed using a number of different tissues and sequencing technologies. Gill transcriptomes of heat-resistant and non-resistant strains of trout were compared in [33,34] to explain the mechanisms the former use to survive high temperature. Rebl et al. [33] found an increased expression of HSP90, serpin protease inhibitor H1 (aka HSP47), and cytochrome P450-dependent monooxygenases in both strains, confirmed by qPCR. Narum and Campbell [34] also show the expected effects on genes known to be heat-regulated, including HSPs, NF-κB inhibitor, and cytochrome P450. It is also interesting to note that, in this work, there are more differentially expressed genes in heat-acclimated fishes, suggesting either a complex heat response in them, or an overall metabolic shutdown in non-acclimated specimen preventing the activation of physiological stress response. A large number of transcriptomic studies have been performed using kidney tissue [35,36,37,38]. Verleih et al. [35] note the expected increase in HSPs and a number of antioxidant enzymes, and not quite expected absence of cell growth arrest. For a heat-resistant BORN strain, they also note the modulation of cytoskeleton production and lipid metabolism, presumably meant to keep those systems homeostatic under changing conditions. Similar results were produced in [36]: more HSPs, less overall metabolic activity, slightly upregulated spliceosome activity and immune responses. Zhou et al. [38] also note cytokine/chemokine involvement in heat response. Liver is also a common object of study in trout heat response [39,40,41]. The first work detected the increased expression of HSPs (HSP90, HSP47, HSP40), as well as noting a number of metabolic processes, DNA replication initiation and acute inflammatory response among significantly affected GO terms/KEGG pathways. Quan et al. [40] were mostly concerned with lncRNAs as regulators of heat stress response, but they have also analyzed the targets of those regulators and detected suppression of a number of metabolic pathways. They have also measured several ROS-related parameters (superoxide dismutase and catalase activities, glutathione peroxidase content and malondialdehyde content) and found all of them significantly increased in heat-stressed fishes, further confirming the role of ROS and other free radicals in heat stress. Finally, there is a single work [42] centered on the response of nucleated red blood cells to acute heat stress (3 °C per hour temperature increase until 25 °C) and post-stress recovery (a return to normal temperature after 1 h at 25 °C) with sampling 4 h and 24 h after exposure. The set of genes upregulated shortly after heat treatment includes HSPs and HSP-organizing protein, as well as a number of transcriptional factors, such as NF-κB inhibitor alpha, AN1-type zinc finger protein, and jun-B. The increased expression of NF-κB inhibitor probably facilitate the maintenance of the intracellular pool of NF-κB indicating the involvement of NF-κB signaling in stress response. A similar upregulation was previously shown in trout after the experimental activation of NF-κB signaling [43]. NF-κB is mostly anti-apoptotic, and all three factors are commonly implicated in stress response, although no direct conclusions on the processes they potentially regulate were provided [42]. Although both high-temperature response in trout and response of animal cells to taxifolin were studied separately, there are no detailed studies on combined taxifolin/heat treatment in trout. Thus, the goal of our work was to assess taxifolin efficiency as a heat-stress mitigating agent, and to shed some light on the effects that such a combined treatment has on fish.

## 2. Materials and Methods

### 2.1. Experimental Diet

The commercial diet EFICO Alpha 717R (BioMar, Denmark), containing 22–25% lipid, 40–43% protein, 20–23% carbohydrate, 2.8–5.8% fiber, 0.9% total P, 4–7% ash, and 22–25 MJ kg^−1^ total energy, without any supplements, was used as the basal diet. The experimental diet was made by supplementing the basal diet with taxifolin (1000 mg kg^−1^ of diet). Taxifolin (quality and safety certificate no. 396-08.17) was purchased from Ametis (Russia). Distilled water was used to dissolve supplement; then, the solution was heated up to 45 °C and stirred continuously for 1 h. Visual control of the dissolution process made it possible to make sure that there was no sediment. The solution with supplements was sprayed onto feed pellets from a spray gun directly on the day of feeding. Then, the feed pellets were dried at room temperature for an hour. In the control group, distilled water alone was added to the feed.

### 2.2. Fish Rearing

On 6 December 2019, rainbow trout, *O. mykiss*, juveniles (0+ age, 66.8 ± 15.7 g average body weight, 17.4 ± 1.6 cm average body length) obtained from a commercial trout farm (Ladmozero Lake, Republic of Karelia, Russia) were transported to the Laboratory of Environmental Biochemistry at the Institute of Biology, Karelian Research Centre of the Russian Academy of Sciences (Petrozavodsk, Russia). Fish were randomly stocked into 8 glass tanks (250–270 L capacity) with 20–21 fish per tank. Tanks were continuously supplied with aerated water with the flow rate set at 0.16 L min^−1^, water temperature 14 ± 1 °C, dissolved oxygen 7.5–8.5 ppm, total ammonia nitrogen < 0.1 mg L^−1^, nitrite nitrogen < 0.1 mg L^−1^, and nitrate nitrogen < 10.0 mg L^−1^, under natural photoperiod. During the acclimation period, all fish were given the basal diet.

### 2.3. Experimental Set-Up and Sampling

On 22 January 2020, at the start of the experiment, acclimated fish were divided into four groups (two tanks per group) as follows (HS—heat stress, T—taxifolin): HSnoTno, HSyesTno, HSnoTyes, HSyesTyes. The fish were fed one of two diets: a basal diet without any supplements (groups HSnoTno and HSyesTno) or a basal diet supplemented with taxifolin (1000 mg kg^−1^ of diet; groups HSnoTyes and HSyesTyes). The fish were fed once a day; the feeding level based on percent of tank biomass was equal for all groups. The food was eaten completely. On 22 February 2020, after 1 month of the experimental diet, the groups HSyesTno and HSyesTyes were treated with heat stress by using a temperature system that could continuously increase the temperature at a constant speed (increments of 1 °C per 48 h). The temperature change was considered to approximately simulate the natural temperature increase in cage culture waters. Early morning of 10 March 2020, all fish from one tank (tank no. 3, group HSyesTno) died. It happened when the water temperature in tanks nos. 3, 5 (group HSyesTno) and nos. 4, 7 (group HSyesTyes) reached 23 °C and was maintained for 24 h. No deaths occurred in other tanks. The dissolved oxygen level in tanks nos. 3, 5 (group HSyesTno) and nos. 4, 7 (group HSyesTyes) decreased from 7.5–8.5 (22 February 2020) to 3.7–4.2 ppm (10 March 2020). We decided not to increase the aeration for groups exposed to heat stress and not to maintain similar dissolved oxygen levels for all groups. The coincidence of an increase in water temperature and a decrease in dissolved oxygen content is typical for natural reservoirs.

Then, (daytime of 10 March 2020) the remaining fish were anesthetized with a lethal dose of clove oil before length and weight measurements and tissue sampling (Table 1). Liver samples for transcriptome analysis were collected immediately, flash-frozen in liquid nitrogen, and stored in liquid nitrogen until RNA extraction.

### 2.4. Gyrodactylus sp. Infections

At the beginning of February, all fish from all tanks showed the first signs of a parasitic disease such as increased mucus secretion, skin lesions, eroded fins, and reduced activity and appetite (deaths were not recorded). On 15 February 2020, eight fish (one fish per tank) were randomly selected and examined to determine their ectoparasite status using a stereomicroscope LOMO MSP-2 (45×). The disease was identified as gyrodactylosis. At the end of February 10, fish from each tank were sacrificed for further biochemical analysis. As the stocking density decreased, the welfare of rainbow trout improved, and the visual manifestations of the disease disappeared, so no specific treatment was given.

When sample collection (10 March 2020) all fins from each fish were placed in tubes filled with 96% ethanol to count the number of *Gyrodactylus* sp. individuals (Table 1).

### 2.5. RNA Isolation, Library Preparation, and Sequencing

Total RNA was extracted from the liver samples using the standard PureZOL protocol (Bio-Rad, Hercules, CA, USA), and the RNA purity was checked using the Implen NanoPhotometer C spectrophotometer (Implen, Munich, Germany) and agarose gel electrophoresis. cDNA libraries were prepared using The NEBNext Poly(A) mRNA Magnetic Isolation Module (E7490) (NEB, Ipswich, MA, USA) and NEBNext Ultra II Directional RNA Library Prep Kit for Illumina (E7760) (NEB, Ipswich, MA, USA), according to the manufacturer’s instructions. Index adapters were added to identify sequences for each sample in the final data (NEBNext Multiplex Oligos for Illumina (Dual Index Primers Set 1, E 7600) (NEB, Ipswich, MA, USA). Subsequently, the 20 libraries were subjected to paired-end sequencing on the Illumina HiSeq 2500 platform.

### 2.6. Data Analysis

After quality control with FastQC [44], the reads were mapped to a published *O. mykiss* assembly [45] using HiSat2 [46]. After converting mapping results to per-gene read counts with SAMtools featureCounts utility [47], the analysis of differential expression was performed in R. DE genes were identified using EdgeR Quasi-Likelihood pipeline [48,49] (weight algorithm, Fisher exact test) at an FDR cutoff of 0.05 and absolute log-fold change cutoff of 1. Differentially expressed genes were identified for all four possible permutations of temperature/taxifolin contrast, as well as between fishes with high and low parasite loads (using ad hoc cutoff of 100 *Gyrodactylus* specimen per fish) within the no heat stress/no taxifolin group. GO term enrichment was performed for all contrasts with topGO R package [50] and visualized with GOplot [51].

As library 120 was found to be different from all other libraries in GC composition, as well as exhibiting a pattern of expression dissimilar to other samples within its group, DE gene identification and GO enrichment analysis were performed separately on a complete dataset and on a dataset excluding this library. To study the anomaly within this library, it was independently assembled de novo with Trinity [52]. The produced contigs were BLASTed against the *O. mykiss* assembly. The reads from this library were also mapped to SILVA 138.1 SSU rRNA reference alignment [53] with Mothur [54] to estimate the amount of rRNA-derived reads.

## 3. Results

### 3.1. DEGs and Possible Artifacts

**Figure 1:**
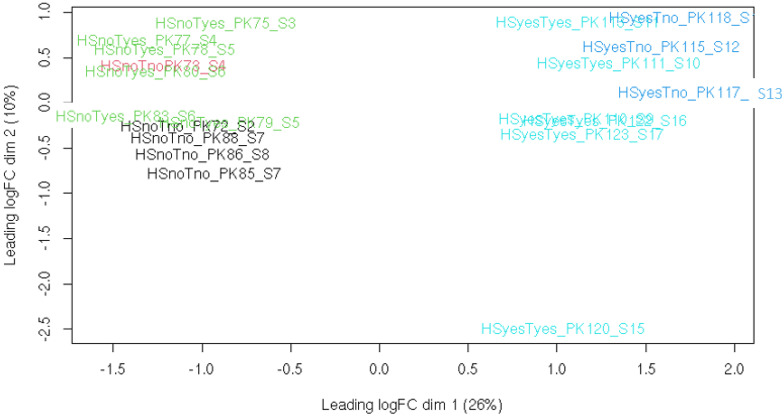
Principal component analysis plot for all libraries including specimen 120. Treatment groups are color−coded.

To obtain a general overview of libraries, PCA was performed on per-gene read counts (Figure 1). It is clear from this plot that heat stress is a major source of variation between samples, with the presence or absence of taxifolin a less significant factor, and parasite load having no obvious effect at all. Specimen 120 appears to be an outlier: the distance between this fish and all others is roughly similar to that between samples with and without heat stress. Its exclusion does not affect the relative positions of other libraries (PCA plot with this library excluded is available as Figure S1).

During the read quality check, this library was also shown to have a GC-content distribution dissimilar to all other libraries, and it appears to be distinct from other libraries collected in similar conditions on heatmaps (Figures S2–S4). To control for possible contamination with non-trout mRNAs, this library was independently assembled de novo with Trinity. The resulting scaffolds (106391, including isoforms) were mapped to *O. mykiss* assembly with BLAST. Only 1877 (~1.7%) failed to produce at least one hit with 80% or more identity, and the majority of these sequences are either low complexity or produce other teleosts as top BLAST hits against NCBI nr. This result does not support large-scale contamination with mRNAs of some distant organism (such as *Gyrodactylus* worms present in studied fishes). To identify the possible rRNA contamination, the complete library was mapped to SILVA SSU rRNA alignment with Mothur; however, the median alignment length was 10 nucleotides, and the 97.5th percentile was 43, suggesting that rRNA reads comprise under 10% of the library. Additionally, even if rRNA abundance is underestimated, there is no plausible mechanism by which rRNA contamination would affect the read counts for protein-coding genes.

Thus, neither foreign mRNA nor rRNA appear to be present in sufficient amounts to explain the observed difference between library 120 and others. The specimen itself also is not remarkably different from other fishes used in this study (Table 1). Without a clear explanation of the peculiarities in library 120 (and thus without being certain whether it is compromised or not), we have performed the search for DEGs and enriched GO categories both including and excluding this library. The study design allows for four different contrasts: heat shock effects in the presence or absence of taxifolin, and the effect of taxifolin in the presence or absence of heat shock. As it turned out, the addition of taxifolin without heat stress did not produce any statistically significant differences in gene expression; the numbers of differentially expressed genes for other three contrasts are shown in Table 2.

**Figure 2:**
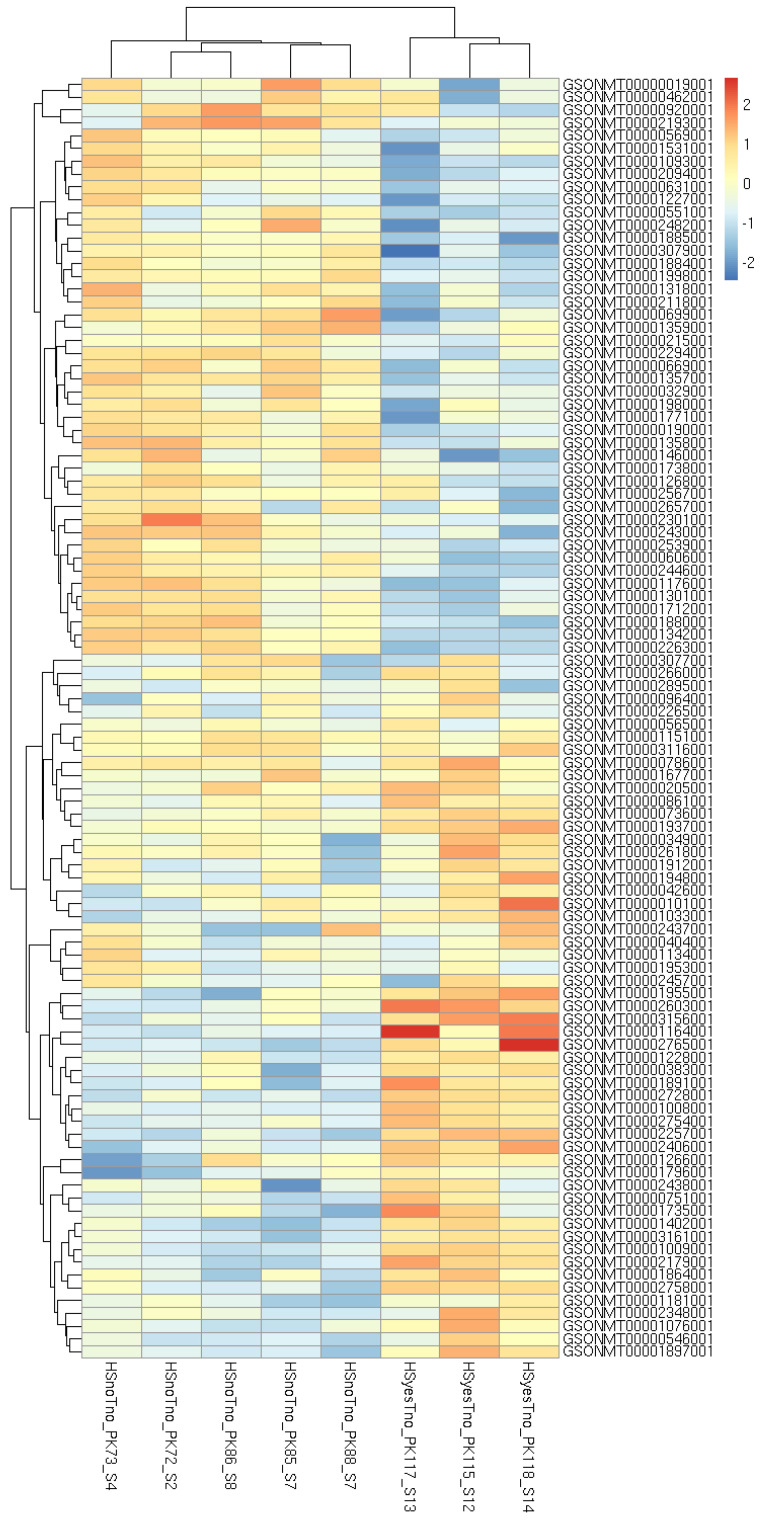
Heatmap of 100 most significant differentially expressed genes affected by increased temperature without a taxifolin treatment.

**Figure 3:**
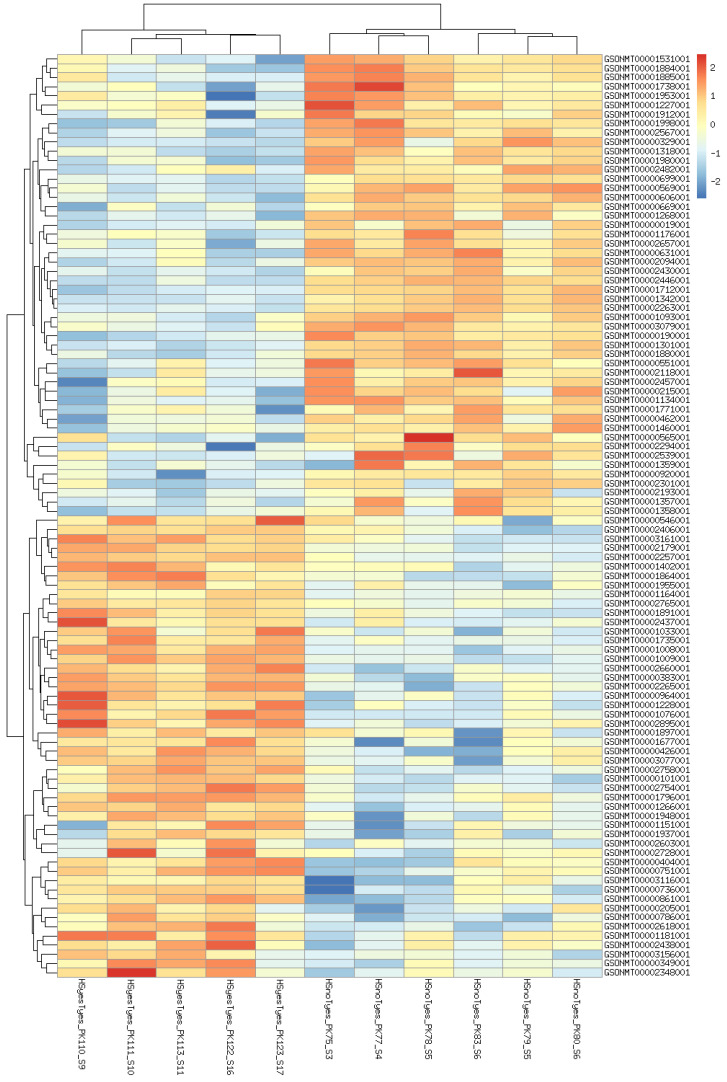
Heatmap of 100 most significant differentially expressed genes affected by increased temperature in taxifolin−treated fishes.

**Figure 4:**
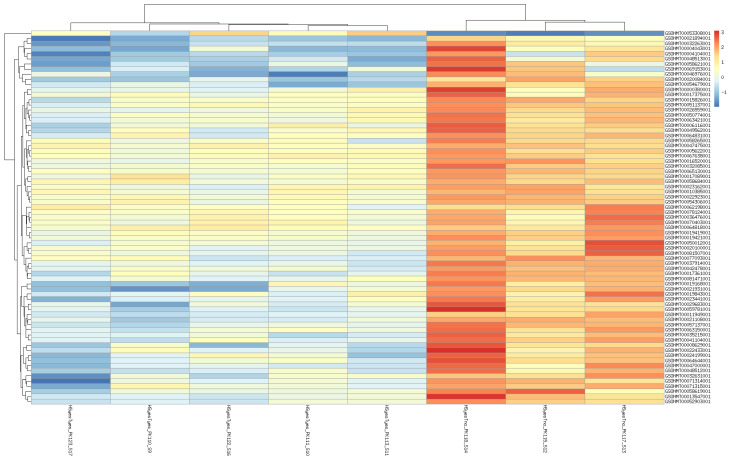
Heatmap of differentially expressed genes affected by taxifolin treatment under increased temperature.

Although specimen 120 belongs to the HSyesTyes group, its inclusion or exclusion affects the HSyesTno/HSnoTno contrast, presumably by increasing (dataset-wide) variance and thus the threshold of significance. For other two contrasts, its exclusion decreases the number of identified DEG, although the results of the two analyses largely overlap. Further, the difference mainly comes from genes with relatively high adjusted *p*-values, often very close to significance threshold (Figure S5). The analyses including specimen 120 produced fewer significantly enriched GO categories, although most of the categories remained enriched in either analysis (Figures S6 and S7). Since this library is a clear outlier, and its inclusion does not produce highly significant DEGs, we have decided to exclude it from further analyses. Top 100 differentially expressed genes (by FDR) in all contrasts are shown on heatmaps (Figure 2, Figure 3 and Figure 4). Per-gene read counts for all libraries including specimen 120 are available in Table S1; complete lists of differentially expressed genes used in Figure 2, Figure 3 and Figure 4 and further discussion are available in Tables S2–S4.

Although not intended by the study design, fishes within all eight aquaria were contaminated with *Gyrodactylus* sp. The parasite load varied from fish to fish (Table 1), although it tended to be lower in the heat stress treatment group, presumably because high temperatures are detrimental for the parasite as well as for its host. To estimate whether it could affect the results of the study, we have attempted to identify the genes differentially expressed between fishes with high and low parasite load (defined as those with more or less than 100 parasites per fish). Since the parasite load correlates with temperature, an analysis of the whole dataset would be heavily confounded by effects of heat. Out of four available groups, only the HSnoTno group had more than one fish on either side of parasite contrast, so a search for potential parasite-associated DEG was limited to this group. Using the same cutoffs as in other comparisons (FDR ≤ 0.05, abs(logFC) > 1), we detected no genes whose expression is significantly affected by parasite load. The PCA of all transcriptomic libraries (Figure 1) also does not show any clustering caused by parasite load, except what could be explained by temperature effect.

This result does not necessarily imply that *Gyrodactylus* infection has no effect on trout transcriptome: it is likely that comparing infected and non-infected *O. mykiss* specimens would identify the response to *Gyrodactylus*. However, the parasite load does not appear to be a significant confounder for intended comparisons.

### 3.2. GO Enrichment

The addition of taxifolin to feed has no statistically significant effect on gene expression in trout liver under normal conditions. Its direct effect under heat stress, as measured by a comparison of expression patterns between taxifolin-fed and taxifolin-free fishes under heat stress, is also relatively small (74 differentially expressed genes, no significantly enriched GO BP categories). However, this comparison may be underestimating the degree to which taxifolin modulates heat stress response. There is overlap between heat-responsive genes in taxifolin-fed and taxifolin-free fishes, but shared genes comprise less than half of DEGs in either group.

**Figure 5:**
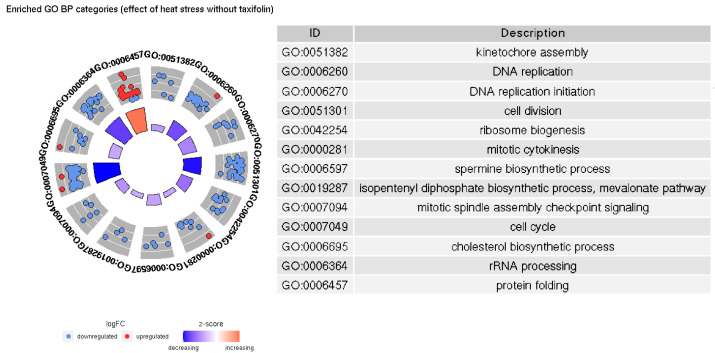
Significantly enriched GO BP categories in the HSnoTno/HSyesTno contrast.

**Figure 6:**
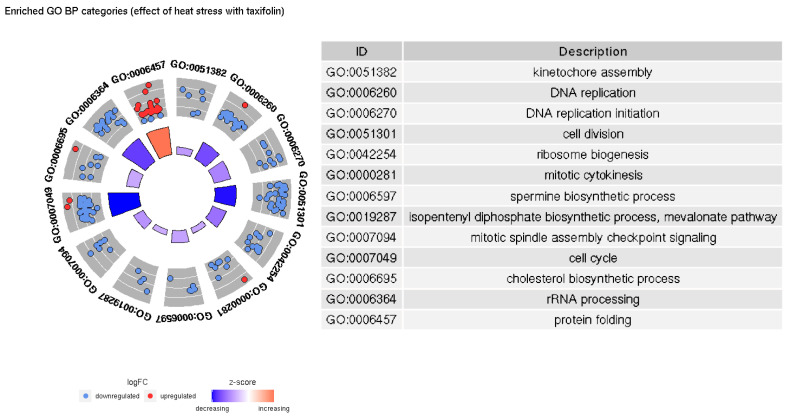
Significantly enriched GO BP categories in the HSnoTyes/HSyesTyes contrast.

A general overview of the processes affected in either group is given by GO term enrichment results (Figure 5 and Figure 6).

In both groups, increased temperature causes upregulation of protein folding-related genes and downregulation of genes involved in DNA replication and cell division; however, there are more significantly enriched terms and genes in taxifolin-treated fishes. Our results implying downregulation of cell growth and the activation of heat shock proteins in trout liver due to heat stress are similar to those described in a previous study [39]. However, we do not observe a significant increase in inflammation- and cell death-related categories. Inflammatory processes were documented among the major differentiated GO terms in trout under heat stress [39]. Lewis et al. [42] have also noted the increased expression of kappa-B inhibitor after the stress, presumably to recover the pool of this protein depleted by NF-κB activation. A relatively weak upregulation of kappa-B inhibitor alpha is present in HSyesTyes/HSnoTyes contrast (LFC 1.05, FDR 0.0009), but GO enrichment does not support a significant inflammation-related response. Further, no differential expression of interleukins, TNF- α or most Nod-like receptor genes was observed. The only DE NOD-like receptor gene (GSONMT00026925001) is downregulated in HSyesTno/HSnoTno contrast (LFC −2.01, FDR 0.03).

## 4. Discussion

### 4.1. Enriched GO Categories with and without Taxifolin

The major difference between the GO BP categories enriched in HSyesTyes and HS yesTno/HSnoTno contrasts is that a number of biosynthetic processes (specifically, cholesterol, spermine, and isopentenyl diphosphate) only appear to be significantly downregulated in the former, i.e., in the presence of taxifolin. Spermine is a polyamine involved in a number of processes including the redox response, the regulation of transmembrane ion channels, conformational changes in various macromolecules, and the expression of multiple genes [55]. It is also capable of directly scavenging free radicals and thus preventing DNA damage [56]. However, the downregulation of spermine synthesis is not necessarily in response to taxifolin decreasing ROS concentrations: first, the overall difference in ROS-responsive genes between taxifolin-treated and control fishes is relatively weak (see below). Second, the statistical significance of term enrichment may not imply the biological difference. Three out of five genes annotated with this GO pathway are downregulated both in HSyesTyes/HSnoTyes and HSyesTno/HSnoTno contrasts with comparable LFCs, and the other two are their paralogs, so the synthesis of spermine is probably affected by heat stress regardless of taxifolin treatment.

As for the cholesterol and isopentenyl diphosphate (IDP) biosynthesis pathways, it is reasonable to consider them together because the latter compound is a precursor to the former. Further, all genes annotated as part of IDP biosynthesis pathway (GO:0019287) are also annotated as a part of cholesterol biosynthesis pathway (GO:0006695), so the enrichment of these two pathways is a result of the downregulation of the same set of genes. Cholesterol biosynthesis is also the only significantly enriched GO category in GO enrichment analysis on genes that are only DE in HSyesTyes/HSyesTno contrast. In combination with heat stress, taxifolin downregulates the majority of isoprenoid pathway enzymes, including HMG-CoA synthase and reductase, mevalonate kinase, and diphosphomevalonate decarboxylase. In the downstream chain of reactions converting IDP to cholesterol [57], only delta(14)sterol reductase is affected.

Taxifolin is known to affect cholesterol concentration in liver tissues and blood serum or shift HDL/LDL cholesterol ratio, giving this compound a potential role in atherosclerosis prevention and treatment. There are multiple models attempting to explain the interactions between taxifolin and cholesterol (see [10,11] for a detailed review). According to one of them, taxifolin directly removes myeloperoxidase-produced NO radicals that would otherwise lead to lipid peroxidation of LDLs (and, hypothetically, HDLs), thus affecting their ratio [58]. It can also affect the HDL/LDL ratio by altering apoA/apoB formation and thus altering the dynamics of cholesterol secretion and uptake [23,59]. Finally, it decreases cholesterol production in hepatic cell cultures by inhibiting HMG-CoA activity, a rate-limiting step of IDP synthesis [23]. Our results suggest that the latter effect is produced by downregulating HMG-CoA reductase expression (as well as that of other enzymes in the pathway) rather than the direct inhibition of enzymatic activity by taxifolin. It is also important to note that IDP is a precursor to multiple sterols and steroids other than cholesterol, suggesting that other physiological effects of taxifolin may be mediated by their shortage.

### 4.2. Other Processes Affected by Taxifolin Treatment and Heat Stress

Although not present among the significantly enriched GO terms, a number of processes and protein families are known to be affected by either taxifolin or heat stress. Among the latter, heat shock proteins are the most obvious group of interest. It is also important to note that besides its direct effects in protein folding, the HSP70 family also plays a role in downregulating the expression of pro-inflammatory genes [60,61], so any effect that taxifolin has on inflammation may be (at least partly) mediated by HSP70 activation.

The diversity of HSP70/110 and HSP90 families in trout was studied in recent papers ([37,62], respectively), which we use as a reference for identifying individual family members. The upregulation of these families was documented in most transcriptomic studies on heat stress in rainbow trout (as well as other animal models), and it is no surprise that they also display upregulation in our data for both taxifolin-treated and control fishes. The only exception is hspa4L, some homologs of which are downregulated in HSnoTyes/HSyesTno comparison. For most members of the heat shock protein family, log-fold change is lower under taxifolin treatment, i.e., although they are overexpressed under heat stress regardless of taxifolin presence, this effect is less significant in fishes exposed to the compound (Table S5). The difference is especially pronounced in HSP70 sensu stricto (hsp70a and hsp70b), where raw expression differences reach as much as an order of magnitude. The ROS-dependent expression of this gene was previously shown in human and animal models (see [63] for a review), suggesting that the antioxidant activity of taxifolin prevents the upregulation of HSP70 by sequestering ROS that could trigger HSP70 overexpression. A slight decrease in HSP70 production under antioxidant treatment was previously shown in trout [64], although using protein concentration rather than transcript counts.

Taxifolin affects the expression of HSP47 as well: out of two paralogs annotated in reference assembly, one is not significantly DE in either contrast, while the other shows slightly weaker upregulation under taxifolin treatment. HSP90s follow the same pattern with some of the genes being very responsive to taxifolin (not DE vs. 8.04 log-fold change), and others showing slight changes in LFC in either direction.

**Figure 7:**
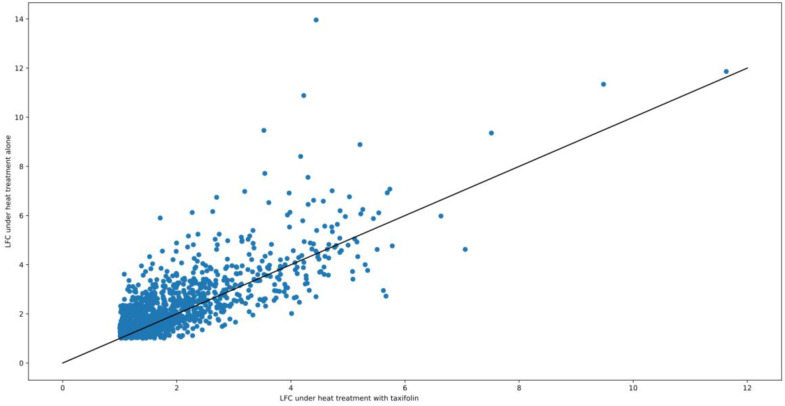
The scatter plot of LFC ratios in HSyes/HSno contrasts with or without taxifolin. Each point corresponds to a gene; the genes that are not DE in one of the contrasts are not included in this plot.

It is important to note that apparently lower LFC values in taxifolin-treated fish under heat treatment do not necessarily imply downregulation of corresponding genes (or their regulators) by taxifolin. Antioxidant treatment was previously shown to increase the production of Nrf2 and heme oxygenase in rainbow trout even without any stress treatment [64]. Thus, it is possible that increased transcript abundance in fish treated with taxifolin alone, rather than weaker upregulation under combined heat/antioxidant treatment, drives the decrease in LFC. This trend of weaker LFC under taxifolin treatment is not limited to HSPs: on average, absolute values of LFC under heat treatment are higher in the taxifolin-free group for most of the genes that are differentially expressed (Figure 7).

Both taxifolin and heat stress are known to affect the antioxidant system of the cell [14,40,65], and such changes are observed in our dataset. In particular, glutathione synthase regulatory subunit, one superoxide dismutase gene out of four annotated, and two glutathione peroxidase genes (out of 10 annotated) are upregulated under heat stress both with and without taxifolin. In all cases, LFCs are below 2 and slightly lower under taxifolin treatment, much like HSPs. However, other major elements of ROS response (catalase, thioredoxins and peroxiredoxins, other paralogs of DE antioxidant enzymes) show no differential expression, and one of the glutamate cysteine ligase genes is downregulated under heat stress. NADPH oxidase, an enzyme whose major function is to produce ROS for immune and signaling purposes, is not DE either.

In our opinion, this relatively weak effect of taxifolin on pro- and antioxidant enzyme expression can be interpreted in two major ways. First, the compound could affect the activity of antioxidant proteins without a significant change in their expression. The majority of studies showing the increase in catalase and other antioxidant enzymes in response to taxifolin have relied on direct measurements of enzymatic activity rather than estimates of mRNA abundance, so there is no contradiction between those works and our results. Second, the taxifolin-induced heat resistance could be (mostly) mediated by mechanisms other than its direct antioxidant effect.

Nrf1 and Nrf2 are a family of transcriptional regulators involved in a number of processes, including maintaining the redox balance and cholesterol metabolism [66,67]. The diversity of this family in fish was, to the authors’ best knowledge, never studied specifically, but the trout genome contains two genes annotated as Nrf1. These are differentially expressed in both HSyes/HSno contrasts, with higher LFC without taxifolin. The closest homolog of reference Nrf2 in trout genome, GSONMT00008906001, is not DE in either contrast. Although the activation of these proteins does not directly depend on their own expression levels, increased abundance of Nrf2 transcripts after its activation was observed in a number of works [64,68], presumably to recover the depleted pool of inactive Nrf2 protein.

The Nrf2/ARE pathway was previously implicated in the fish response to taxifolin and other antioxidants (see Introduction), but in our data, not all ARE-associated genes are responsive either to heat stress or taxifolin. Among major ARE substrates known from model objects, many are not DE at all (glutathione synthase catalytic subunit, metallothioneins, NAD(P)H quinone oxidoreductase, inducible nitric oxide synthase). In other protein families, only some of the paralogs are upregulated (ferritin), or two paralogs change their expression in opposite directions (heme oxygenases). One of the genes for glutamate cysteine ligase light chain, a family whose member was directly shown to be ARE-regulated in *Oncorhnychus kisutch* [69], is downregulated rather than upregulated under heat stress, and the other is not DE. On the other hand, glutathione transferases, another ARE-regulated gene, show a clear taxifolin-dependent pattern of activation. Two of the genes in this family are downregulated in HSyesTno/HSnoTno contrast but not in HSyesTyes/HSnoTyes, implying that their downregulation is somehow prevented by taxifolin. Another one is only upregulated in HSyesTyes/HSnoTyes. It is important to note that ARE-regulated genes in fish are not studied as well as those in model objects, so these results do not necessarily disprove the role of Nrf2/ARE in either heat stress response or taxifolin response.

Taxifolin is known to inhibit angiogenesis, which was proposed as one of the mechanisms behind its anticancer activity [11]. Angiogenesis is regulated, among others, by vascular endothelial growth factor (VEGF), which is upregulated in both HSyes/HSno contrasts, although with higher LFC for fishes not treated with taxifolin; it is also downregulated in HSyesTyes/HSyesTno contrast, further confirming that taxifolin downregulates this gene (or, rather, prevents its upregulation). A similar combination of VEGF overexpression with overall cell cycle arrest was observed in hypoxic trout [70], suggesting that perhaps VEGF upregulation is mediated by hypoxia and not directly caused by heat stress. Taxifolin treatment prevents the heat-dependent upregulation of angiopoetin-like protein 4 and the downregulation of plexin. On the other hand, it also prevents the upregulation of trombospondin-1-like, a natural proteinaceous inhibitor of angiogenesis.

Pro-apoptotic activity was also proposed as a mechanism behind the anticancer activity of taxifolin, and apoptosis-related transcripts are indeed affected by a taxifolin treatment in our work. A single homolog of Mcl-1 is differentially expressed in HSyesTyes/HSyesTno. It is weakly downregulated (LFC − 1.04, *p*= 0.01), suggesting that taxifolin shows pro-apoptotic activity by suppressing this negative regulator of the apoptotic process. As for the other two contrasts, neither shows the obvious pro- or antiapoptotic direction of change. In HSnoTno/HSyesTno, we observe the upregulation of another Mcl-1 homolog and the downregulation of IRF1-like protein and caspase 9, which imply antiapoptotic activity. On the other hand, pro-apoptotic CIDE-3, Diablo homolog, and two genes for huntingtin-interacting protein 1-like are also upregulated, which should promote rather than inhibit apoptosis. There is also the upregulation of Bcl2-like protein 2, whose homologs in human may be pro- or antiapoptotic depending on post-translational changes. In HSnoTyes/HSyesTno contrast, NAIF1, Diablo homolog (paralog of the one upregulated in previous contrast), and an unidentified DED domain-containing protein are upregulated, suggesting the induction of apoptosis by heat treatment. On the other hand, another paralog of NAIF1 is downregulated. It is also interesting that all apoptosis-related genes are only DE in one or another of these two contrasts, but not both. In other words, heat treatment shows a completely different effect on apoptosis regulators depending on whether or not taxifolin is present. Although in its presence, there are more pro-apoptotic changes, the effect of taxifolin on apoptosis appears to be more complex than just a simple induction or inhibition.

## 5. Conclusions

As evidenced by the decreased mortality of taxifolin-treated trout under increasing ambient temperature, this compound does provide some degree of heat resistance. Transcriptomic sequencing sheds some light on the processes affected by heat stress with and without addition of taxifolin to the feed, but the exact mechanism behind this resistance remains unclear. Increased viability is unlikely to be explained by antioxidant effects of taxifolin (either directly through radical scavenging or by activating the antioxidant system of fish cells), because the effects of increased temperature on the expression of antioxidant genes do not vary with presence or absence of taxifolin. However, it should be noted that ROS serve as messengers in multiple signaling pathways, so the antioxidant effect could produce (potentially beneficial) metabolic effects even if ROS activity itself is not a major factor in determining the physiological status of a given specimen. Heat shock proteins are also produced in response to increased temperature in both groups, although the increase in their expression is higher in control fishes.

The most pronounced difference in transcriptomic heat response between taxifolin-treated and control fishes is the downregulation of isopentenyl diphosphate production in the former. As this compound serves as a precursor to cholesterol and a range of other sterols, their shortage can have wide-reaching effects on trout physiology, potentially explaining the viability. Apoptosis regulators are also affected in different ways depending on the presence of taxifolin, although in neither group did heat stress produce unequivocal pro- or antiapoptotic effects.

On the other hand, the progress of *Gyrodactylus* infection is not visibly affected by taxifolin. The number of parasites per trout specimen does not significantly vary depending on the presence or absence of taxifolin in the feed. It does vary with temperature, but this is likely due to heat being dangerous for both host and parasite. This infection also does not induce any statistically significant changes in the gene expression of trout grown under temperature optimum (14 °C): no differentially expressed genes were identified between HSnoTno fishes with high and low *Gyrodactylus* counts.

## Figures and Tables

**Table 1:** Length–weight parameters and *Gyrodactylus* sp. infestation of rainbow trout individuals. For groups, the presence of heat stress (HS) and taxifolin (T) is indicated in group name.

No of Tank	Group	No of Fish	Weight, g	Length, cm	Number of *Gyrodactylus* sp. Individuals, n	Transcriptome Sample Name
1	HSnoTno	72	181.4	25.2	1164	PK72_S2
1	HSnoTno	73	203.6	25.8	304	PK73_S4
2	HSnoTyes	75	259.0	35.2	36	PK75_S3
2	HSnoTyes	77	139.3	23.8	631	PK77_S4
2	HSnoTyes	78	183.0	24.7	36	PK78_S5
4	HSyesTyes	110	154.9	24.2	21	PK110_S9
4	HSyesTyes	111	205.4	26.0	48	PK111_S10
4	HSyesTyes	113	107.4	20.1	13	PK113_S11
5	HSyesTno	115	208.2	25.8	20	PK115_S12
5	HSyesTno	117	171.6	25.4	243	PK117_S13
5	HSyesTno	118	150.9	24.2	40	PK118_S14
6	HSnoTyes	79	144.4	23.2	16	PK79_S5
6	HSnoTyes	80	205.3	25.4	48	PK80_S6
6	HSnoTyes	83	136.9	23.6	64	PK83_S6
7	HSyesTyes	120	169.2	25.0	22	PK120_S15
7	HSnoTyes	122	182.6	25.7	33	PK122_S16
7	HSnoTyes	123	187.2	25.0	4	PK123_S17
8	HSnoTno	85	182.3	26.0	72	PK85_S7
8	HSnoTno	86	176.4	25.0	132	PK86_S8
8	HSnoTno	88	106.9	22.0	262	PK88_S7

**Table 2:** The number of differentially expressed genes in various contrasts including or excluding specimen 120.

Contrast	Number of DEGs (Specimen 120 Included)	Number of DEGs (Specimen 120 Excluded)	Overlap
Effect of taxifolin without heat stress	0	0	N/A
Effect of heat stress with taxifolin	2746	2580	2182
Effect of heat stress without taxifolin	2253	2498	2007
Effect of taxifolin under heat stress	68	74	59

## Data Availability

All RNA sequencing data are available at NCBI SRA under Bioproject ID PRJNA823838.

## Supplementary Materials

The following supporting information can be downloaded at: https://www.mdpi.com/article/10.3390/ani12101321/s1, Figure S1: Principal component analysis plot for all libraries including specimen 120. Treatment groups are color-coded; Figure S2: Heatmap of 100 most significant differentially expressed genes affected by increased temperature without a taxifolin treatment (sample 120 excluded); Figure S3: Heatmap of 100 most significant differentially expressed genes affected by increased temperature in taxifolin-treated fishes (sample 120 excluded); Figure S4: Heatmap of differentially expressed genes affected by taxifolin treatment under increased temperature (sample 120 excluded); Figure S5: The distribution of FDR values for differentially expressed genes in two major contrasts; values for genes that are differentially expressed regardless of the inclusion of specimen 120 are shown in black, while those that are only DE with or without this library are shown in red and blue, respectively; Figure S6: Significantly enriched GO BP categories in the HSnoTno/HS yesTno contrast including specimen 120; Figure S7: Significantly enriched GO BP categories in the HSnoTyes/HSyesTyes contrast including specimen 120; Table S1: Per-read gene counts and gene metadata; Table S2: Log-fold changes and statistical significance of all differentially expressed genes for the HSnoTno/HSyesTno contrast; Table S3: Log-fold changes and statistical significance of all differentially expressed genes for the HSnoTyes/HSyesTyes contrast; Table S4: Log-fold changes and statistical significance of all differentially expressed genes for the HS yesTno/HSyes Tyes contrast; Table S5: Differentially expressed heat shock proteins in trout and their respective log-fold changes in the two HSyesTno/HSyesTyes contrasts.
