# Supplementary material for: Taxifolin Modulates Transcriptomic Response to Heat Stress in Rainbow Trout, Oncorhynchus mykiss
Source: Animals (Basel). 2022 May 22;12(10):1321. doi: 10.3390/ani12101321 (PMC9137817; doi:10.3390/ani12101321)
Supplement: Supplementary file 1 [file animals-12-01321-s001.zip › Table S5.pdf]

Table S5: Differentially expressed heat shock proteins in trout and their respective log-fold changes in the two HSyesTno/HSyesTyes contrasts.

**Transcript ID**	**Closest Homolog in [62]**	**LFC in Taxifolin-treated Fish**	**LFC in Control Fish**
GSONMT00004379001	hspa4L	2.39	4.40
GSONMT00015093001	hspa5L (BiP chaperone)	2.05	2.45
GSONMT00015921001	hspa4L	2.07	2.42
GSONMT00018153001	hyou1	1.64	1.29
GSONMT00020100001	hsp70a	4.22	10.88
GSONMT00021034001	hsc70	4.77	4.71
GSONMT00030559001	hspa8b	3.91	3.06
GSONMT00034833001	hspa13	Not significant	2.19
GSONMT00056342001	hspa4L	-2.92	Not significant
GSONMT00056362001	hspa4L 1	-3.44	Not significant
GSONMT00064818001	hsp70a or hsp70b	5.21	8.89
GSONMT00068455001	hsa5 (BiP chaperone)	1.45	1.88
GSONMT00076716001	hspa4L	Not significant	Not significant
GSONMT00077567001	hyou2	2.18	2.6
GSONMT00082713001	hspa4L	1.76	2.91
GSONMT00002889001	hsp47	Not significant	Not significant
GSONMT00020438001	hsp47	5.54	6.11
GSONMT00026598001	hsp90aa	Not significant	Not significant
GSONMT00026599001	hsp90ab	11.63	11.86
GSONMT00028101001	hsp90ba	1.67	1.59
GSONMT00037918001	Grp94	4.66	4.27
GSONMT00079391001	hsp90ba	1.78	2.33
GSONMT00080902001	hsp90aa1	4.86	6.19

GSONMT00080903001	hsp90ab1	9.48	11.34
