# Supplementary figures and images for: Taxifolin Modulates Transcriptomic Response to Heat Stress in Rainbow Trout, Oncorhynchus mykiss
Source: Animals (Basel). 2022 May 22;12(10):1321. doi: 10.3390/ani12101321 (PMC9137817; doi:10.3390/ani12101321)

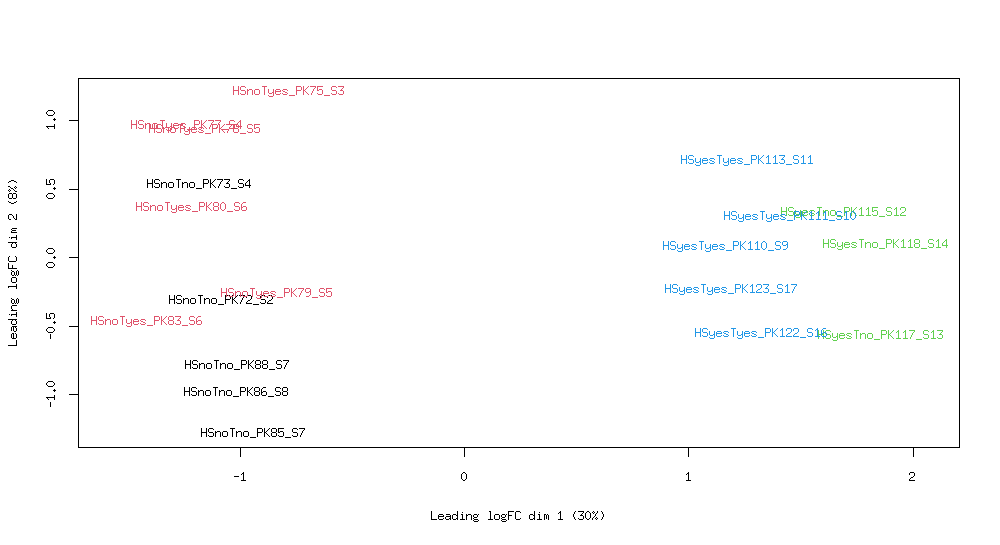

Supplement: Supplementary file 1 [file animals-12-01321-s001.zip › Figure S1.png]

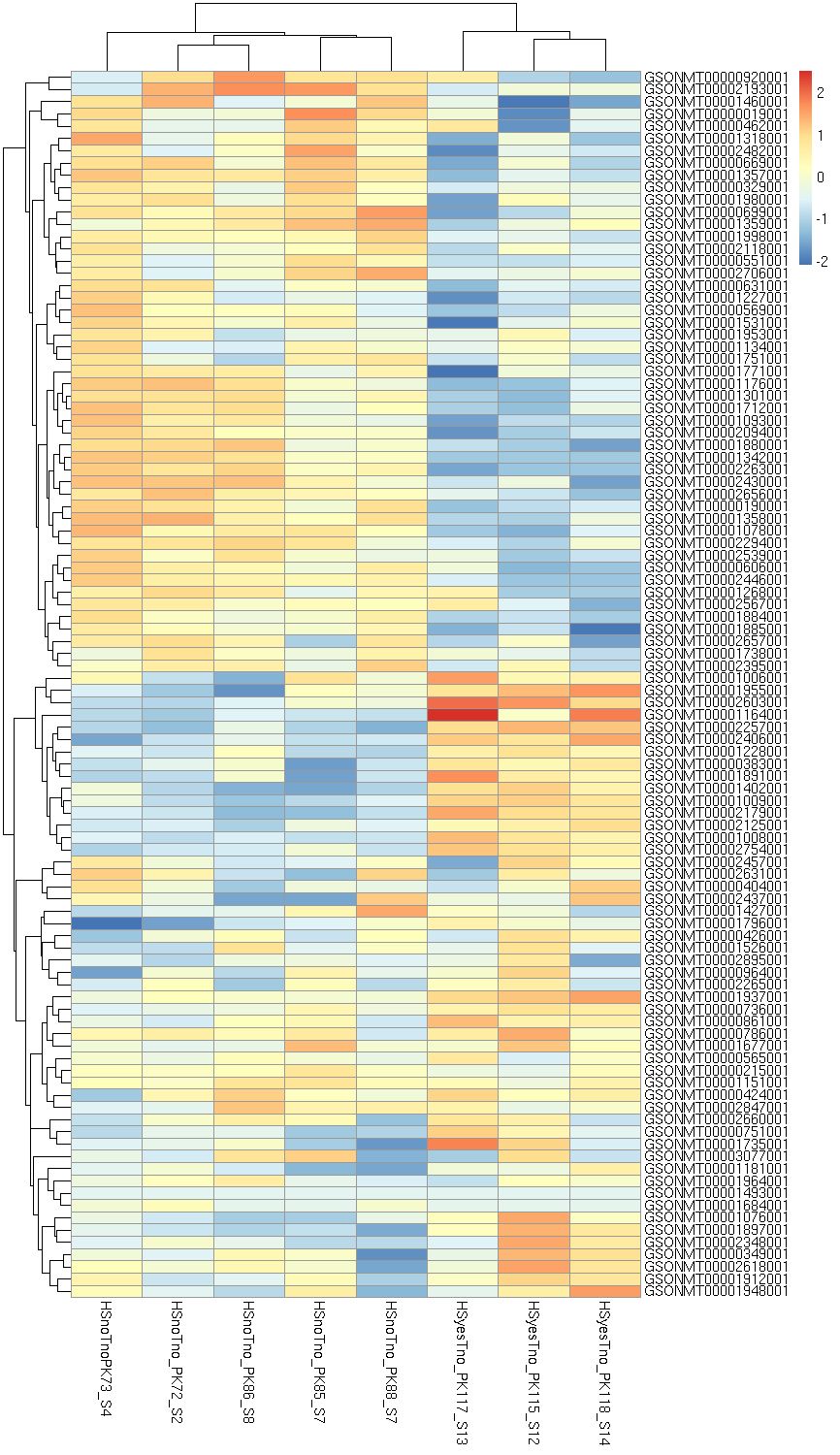

Supplement: Supplementary file 1 [file animals-12-01321-s001.zip › Figure S2.png]

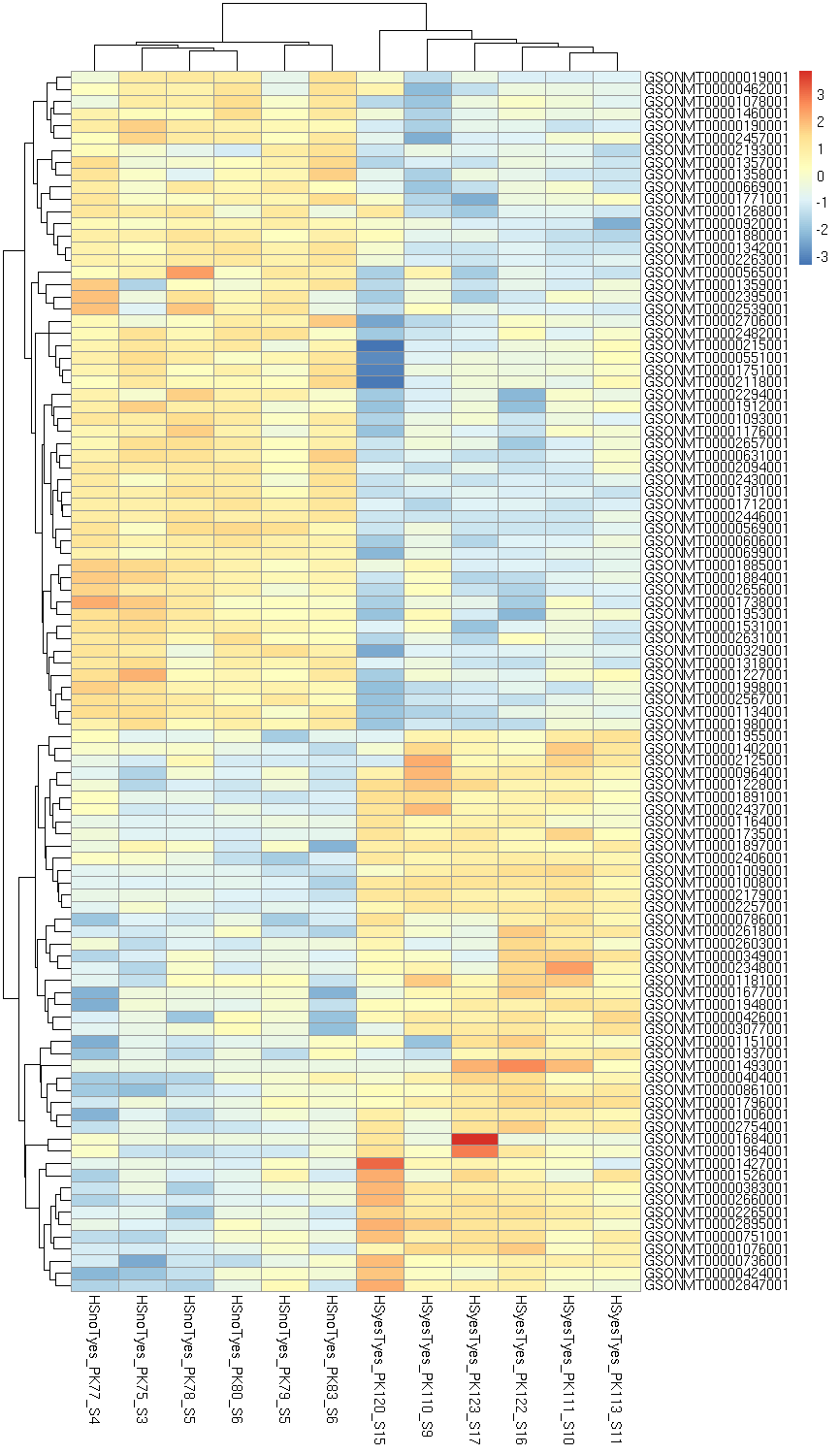

Supplement: Supplementary file 1 [file animals-12-01321-s001.zip › Figure S3.png]

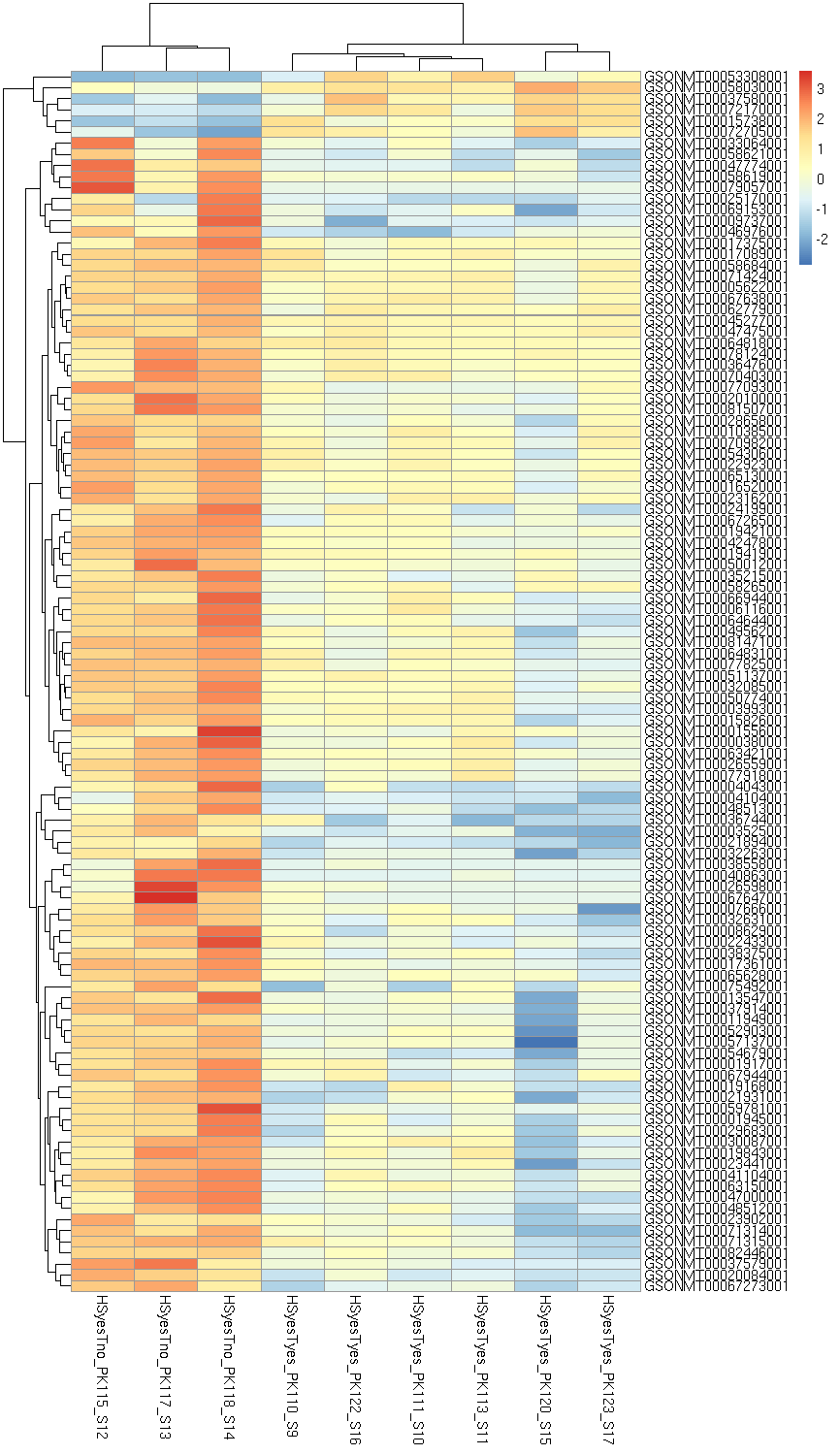

Supplement: Supplementary file 1 [file animals-12-01321-s001.zip › Figure S4.png]

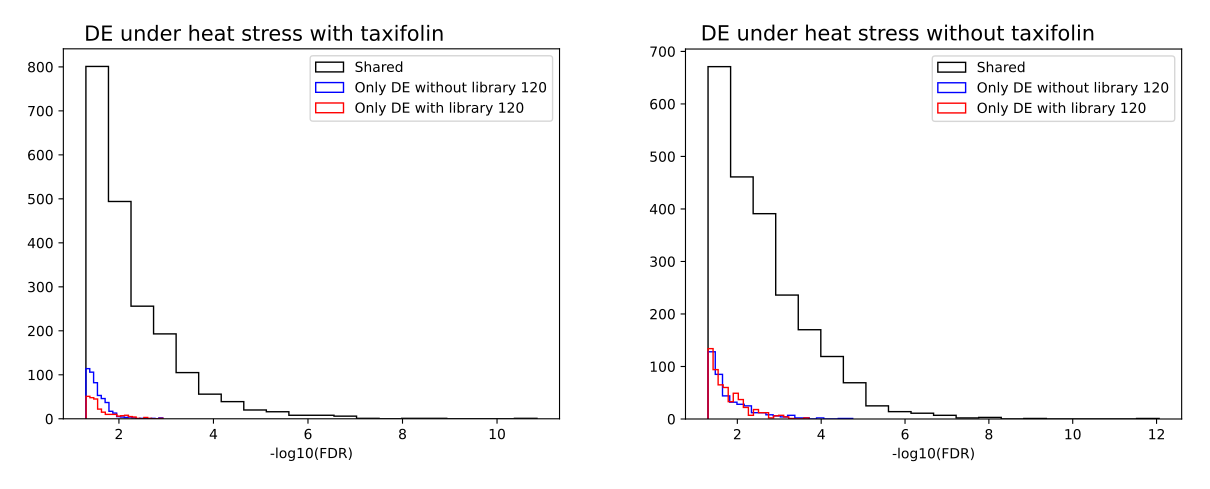

Supplement: Supplementary file 1 [file animals-12-01321-s001.zip › Figure S5.png]

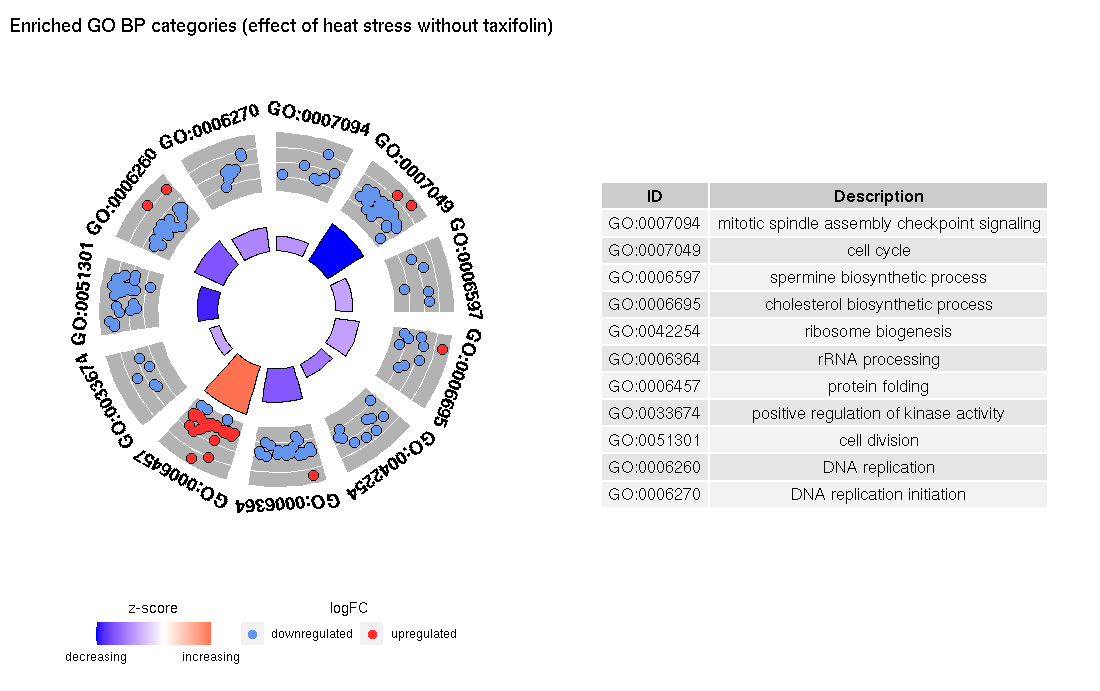

Supplement: Supplementary file 1 [file animals-12-01321-s001.zip › Figure S6.png]

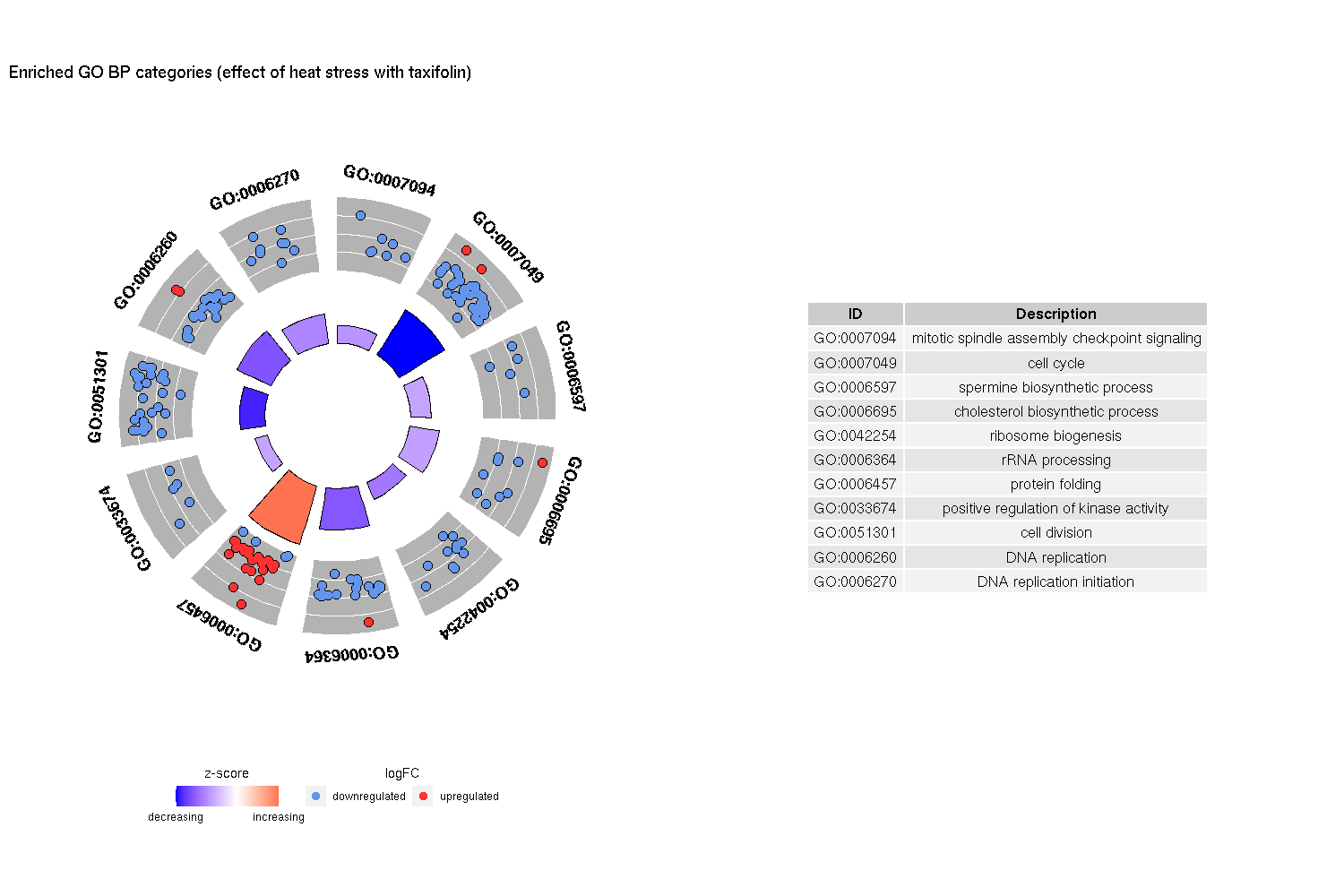

Supplement: Supplementary file 1 [file animals-12-01321-s001.zip › Figure S7.png]
